# Comparison of MRI-based automated segmentation methods and functional neurosurgery targeting with direct visualization of the Ventro-intermediate thalamic nucleus at 7T

**DOI:** 10.1038/s41598-018-37825-8

**Published:** 2019-02-04

**Authors:** Elena Najdenovska, Constantin Tuleasca, João Jorge, Philippe Maeder, José P. Marques, Timo Roine, Daniel Gallichan, Jean-Philippe Thiran, Marc Levivier, Meritxell Bach Cuadra

**Affiliations:** 1 Centre d’Imagerie BioMédicale (CIBM), University of Lausanne (UNIL), Lausanne, Switzerland; 2 Department of Radiology, Lausanne University Hospital (CHUV) and University of Lausanne (UNIL), Lausanne, Switzerland; 3 Department of Clinical Neurosciences, Neurosurgery Service and Gamma Knife Center, Lausanne University Hospital (CHUV), Lausanne, Switzerland; 4 Signal Processing Laboratory (LTS5), Ecole Polytechnique Fédérale de Lausanne (EPFL), Lausanne, Switzerland; 5 Faculty of Biology and Medicine, University of Lausanne (UNIL), Lausanne, Switzerland; 6 Sorbonne Université, Faculté de Médecine, Paris, France; 7 Assistance Publique - Hôpitaux de Paris, Hôpitaux Universitaires Paris-Sud, Hôpital Bicêtre, Service de Neurochirurgie, Le Kremlin Bicêtre, France; 8 Laboratory for Functional and Metabolic Imaging, École Polytechnique Fédérale de Lausanne, Lausanne, Switzerland; 9 Radboud University, Donders Institute for Brain, Cognition and Behaviour, Nijmegen, The Netherlands; 10 Turku Brain and Mind Center, University of Turku, Turku, Finland; 11 Cardiff University Brain Research Imaging Centre (CUBRIC), Cardiff University, Cardiff, UK

## Abstract

The ventro-intermediate nucleus (Vim), as part of the motor thalamic nuclei, is a commonly used target in functional stereotactic neurosurgery for treatment of drug-resistant tremor. As it cannot be directly visualized on routinely used magnetic resonance imaging (MRI), its clinical targeting is performed using indirect methods. Recent literature suggests that the Vim can be directly visualized on susceptibility-weighted imaging (SWI) acquired at 7 T. Our work aims to assess the distinguishable Vim on 7 T SWI in both healthy-population and patients and, using it as a reference, to compare it with: (1) The clinical targeting, (2) The automated parcellation of thalamic subparts based on 3 T diffusion MRI (dMRI), and (3) The multi-atlas segmentation techniques. In 95.2% of the data, the manual outline was adjacent to the inferior lateral border of the dMRI-based motor-nuclei group, while in 77.8% of the involved cases, its ventral part enclosed the Guiot points. Moreover, the late MRI signature in the patients was always observed in the anterior part of the manual delineation and it overlapped with the multi-atlas outline. Overall, our study provides new insight on Vim discrimination through MRI and imply novel strategies for its automated segmentation, thereby opening new perspectives for standardizing the clinical targeting.

## Introduction

The ventro-intermediate (Vim) nucleus is part of the group of motor thalamic nuclei that, along with the anterio-lateral subdivisions, acts as a relay between the basal ganglia, the cerebellum and the motor cortex^1^. It was initially defined by Guiot from electrophysiological recordings^2^ and it is organized in a somatotopic manner, with the leg-area lying laterally and the face-area medially, measuring 2–4 mm in anterio-posterior, 7–10 mm in dorso-ventral and 4–6 mm medio-lateral^3^.

The Vim is typically used as a target for treatment of drug-resistant tremor in functional-neurosurgery framework, such as deep brain stimulation (DBS), radiofrequency thalamotomy^4–7^ or recently, the alternative minimally invasive techniques, radiosurgery (RS)^8–10^ and High Intensity Focused Ultrasound (HIFU)^11,12^.

The Vim cannot be directly visualized on current magnetic resonance imaging (MRI) sequences that are routinely used in clinical practice. The targeting methods are therefore indirect, employing either stereotactic coordinates or the quadrilatere of Guiot^8,9,13–15^. The quadrilatere of Guiot has been established based upon the Vim definition by Guiot and Albe-Fesard while performing electrophysiological recordings^16^. It is a robust and reliable method and is used both in the field of the reference technique (e.g. DBS) or in RS.

In the RS treatment settings, there is no intraoperative confirmation of the target. However, clinical results have shown a reduction in tremor amplitude, comparable to the reference technique^14^. In fact, good clinical outcome was achieved in more than 70% of the cases^14,17^, using the indirect targeting of the quadrilatere of Guiot. Better clinical outcome is usually correlated with higher 1-year MR signature volumes^18,19^. Radiological hyporesponders are rather exceptional, usually accounting for approximately 20% of patients and are frequently, but not always, related to lower clinical improvement.

Nevertheless, the used targeting techniques, including the quadrilatere of Giout, are built upon stereotactic standard landmarks of the brain, not necessarily in a well-established relationship with the underlying thalamic morphology^2^ and therefore, are not sensitive to inter-subject variability^20^. Additionally, the potential of variability increases with the lack of consensus for a “gold standard” targeting method among the centres. Such a drawback is particularly crucial for Vim RS, which cannot rely on intraoperative target confirmation. Hence, there is a need for an improvement and standardization the targeting procedure.

**Figure 1:**
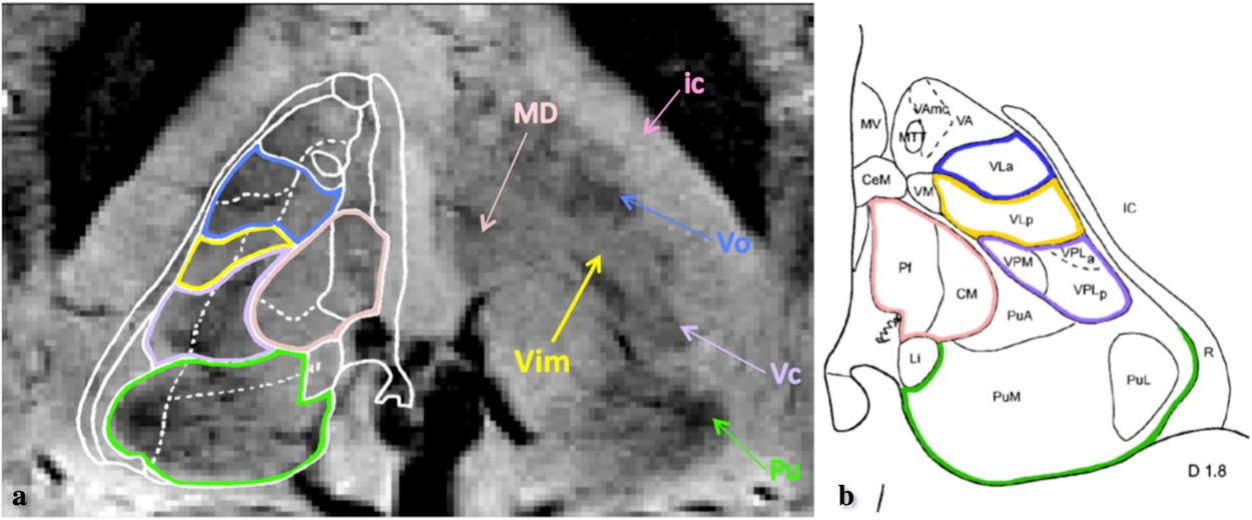
Illustration of the visible structures corresponding to the thalamic area in axial view on the SWI acquired at 7T, based upon the illustration from the pioneering work of Abosch *et al*.^21^. In panel A the SWI features are compared with the Schalterbrand atlas (plate 53 Hd + 3.5) superimposed on the right thalamus. The arrows and the respective color contours indicate the nuclei: Vim, Pulvinar (Pu), ventro-caudalus (Vc), ventro-odalis (Vo), the medio-dorsal group (MD) and the internal capsule (ic). The shown SWI image is part of the dataset used in this study. Panel B gives a corresponding axial plate of the Morel’s atlas where the (same) color (shade) matches appropriate regions of the Shalternbrand’s atlas, while keeping the same nomenclature used in each one of them. In fact, considering the Morel’s atlas^59^ nomenclature, Vim is part of the Ventro-Lateral-posterior nuclei, which furthermore, together with ventro-lateral anterior and ventro-posterior nuclei form the ventral latero-ventral (VLV) group of nuclei.

Advanced MRI techniques applied for acquisitions at ultra-high field could help to address the limitation in Vim-targeting. Recently, Abosch *et al*.^21^ suggested the possibility of a direct Vim visualization by using susceptibility weighted imaging (SWI) acquired at 7 T that showing an enhanced image contrast inside the thalamic area^21,22^ (see Fig. 1).

Susceptibility-weighted imaging (SWI) is a MRI technique particularly sensitive to magnetic susceptibility effects^23,24^. For instance, de-oxygenated blood vessels, as well as the calcium and iron-loaded tissue demonstrate, with respect to the surroundings, strong magnetic susceptibility contrast. Therefore, in such regions, SWI provides an enhanced contrast when compared to standard T1, T2, or T2*-weighted imaging. Aside the increased signal-to-noise ratio that can be traded for improved spatial resolution, ultra-high magnetic field strength, such as 7T, has a superior sensitivity to magnetic susceptibility-related contrasts.

SWI acquired at 7T has already been used for the study of other deep brain structures such as the subthalamic nucleus (STN) and substantia nigra (SN)^21,22,25^. A related technique often used in the context of targeting is the quantitative susceptibility mapping (QSM), where the phase of the MR signal is used to compute a map of the susceptibility distribution. QSM has been mainly explored for a direct visualization of the STN^26–28^, but its contrast variation inside the thalamic area is however limited for discriminating the Vim^27,28^. The existing approach for improving the QSM potential for the visualization of the Vim^29^ requires data to be acquired with subjects’ head in various positions in respect to the magnetic field, which is very demanding in terms of clinical feasibility. Moreover, until now, other than a descriptive study performed by Abosch *et al*., no investigation based on 7T MRI, involved a profound analysis of the thalamic subparts or the Vim in particular.

Other advanced high field MRI techniques have been explored for thalamic nuclei identification. Particularly, diffusion MRI (dMRI) has gained interest in this domain thanks to its ability to depict exquisite microstructural details related to the orientation of the white matter fibres, their coherence and the diffusivity affected by the grey matter (e.g. size of neurons or their arrangement), which are different for each thalamic nucleus. However, with the disadvantage of relatively low spatial resolution, the present standard dMRI techniques are limited in distinguishing small thalamic regions such as the Vim.

**Figure 2:**
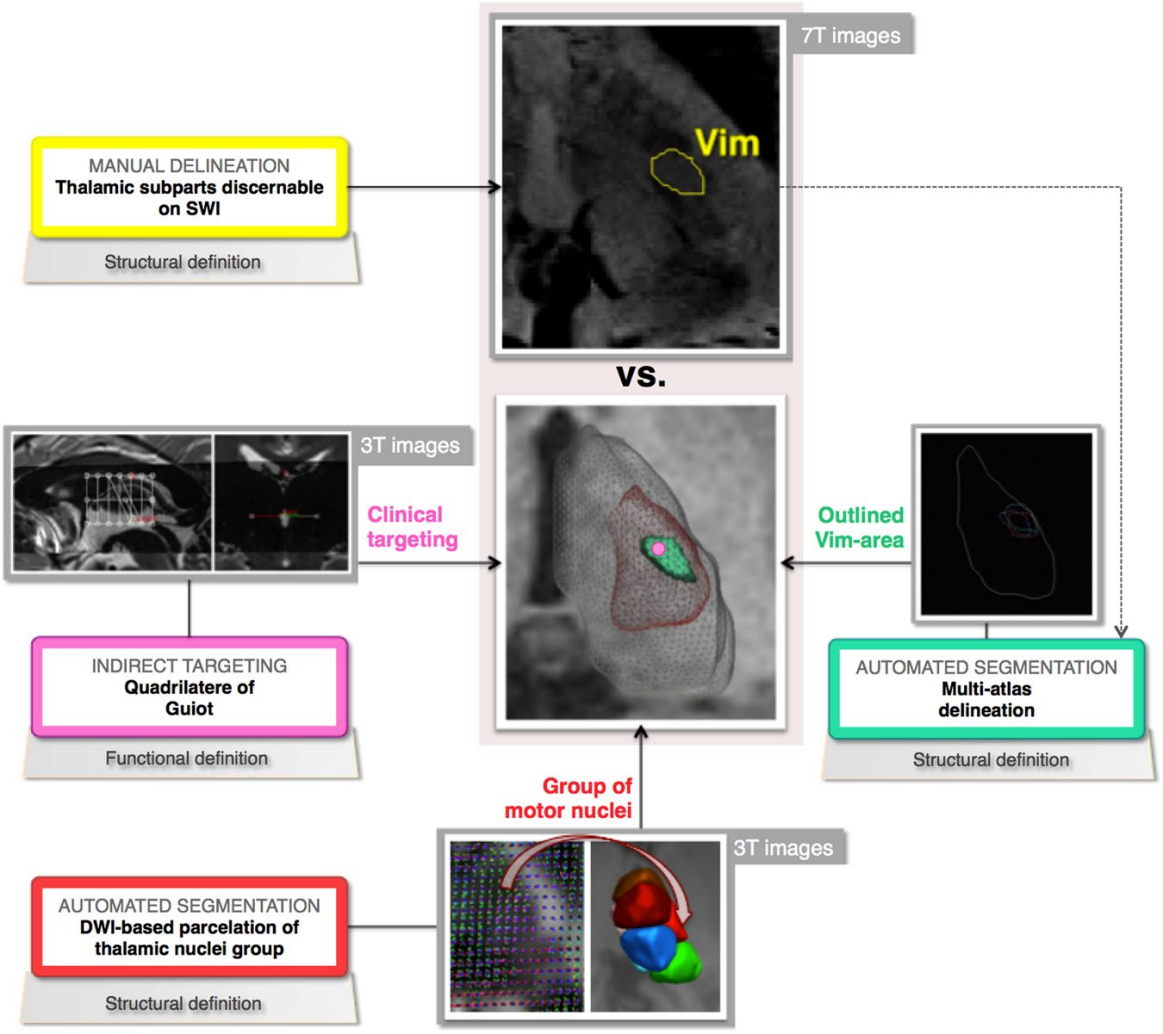
A schematic overview of the performed analysis and comparison in the presented study.

In this study we aim to take a step forward in extended and quantitative analysis of the advantages brought by 7T SWI. Consequently, our goal was a better direct characterization of the Vim. To achieve this, we studied two different types of subjects: a healthy population, in the frame of a preliminary analysis, and a drug-resistant tremor patients’ population, to be able to evaluate the applicability of the previous on real clinical cases. Additionally, in order to assess the performance of the known approaches, the visually distinguishable Vim on 7T SWI will be also compared with three different state-of-the-art methods, namely: (1) the quadrilatere of Guiot, used routinely as clinical targeting and built on 3T data; (2) The automated 3T dMRI-based thalamic subdivision; and (3) The atlas-based Vim outline employing both 3T and 7T data. Each one of these methods is defining the Vim in a different mode. The first method represents the Vim in a functional manner based on statistical findings from previous electrophysiological recordings done on a group of tremor patients. Its outcome is a single point only, that is expected to fall inside the Vim^16^. The remaining two methods, as complementary to the first one, are part of computer-assisted image-analysis techniques and reveal the anatomical structure of the Vim, but differ between them in terms of size of the provided outline. Figure 2 shows an illustrative overview of the performed comparisons.

## Results

Nine healthy subjects without particular brain disease and/or deformation caused by intracranial lesions were scanned at both 3T and 7T. Five of them were relatively young (25+/−2 years old, 2 females, will be referred further as YS1-YS5 respectively) and the remaining four represented the older population (67.2+/−9.5 years old, 1 female, accordingly referred further as ES1-ES4). Please refer to Methods for the acquisition details.

The four approaches investigated for defining the Vim provide outcomes at different sizes: from a single point (e.g. the quadrilatere of Guiot) to a region that encloses a group of nuclei (segmentation from diffusion data). To better assess the spatial distribution, we further divided each region of interest (ROI), either manually or automatically obtained, into eight geometrical sub-regions (refer to Fig. S1 in the supplementary material). More specifically, for the ROI, we calculated the smallest rectangular cuboid containing all non-zero voxels whose mid-plane isolated the superior from the inferior part, while the in-plane diagonals isolated the anterior, lateral, posterior, and ventral parts of the ROI.

The quantitative evaluation of the results included:

- Estimating the intra-subject reproducibility of the Guiot targeting procedures per hemisphere for each subject (each repeated 6 times), by employing the Euclidean distance between the respective points, taking as reference the first targeted point;
- Computing the volumes of all the outlined regions. The volumes were further normalized by the corresponding thalamic volume to ensure a relevant comparison across hemispheres and subjects;
- Assessing the accuracy of the multi-atlas segmentation against the manual delineation done on 7T SWI via the Dice Coefficient^30^ measuring the overlap.

### Manual Vim delineation on 7T SWI

**Figure 3:**
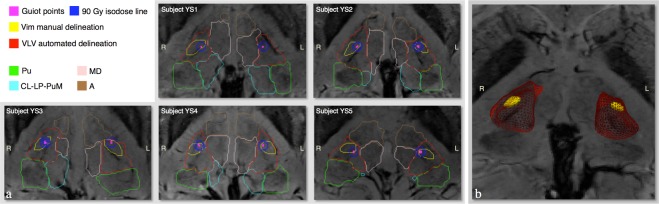
Visual representation of the comparison between the methods. Panel a give the results in axial view of each young subject respectively (YS1-YS5), while panel b shows a 3D view of the Subject YS2’s outlined Vim, as well as its localization inside the VLV cluster and within the thalamus. Among the shown findings, the Guiot points are given in magenta, the manual Vim delineation in yellow and the automatically segmented VLV cluster in red. The remaining automatically delineated clusters shown in panel A are: Pulvinar (Pu), medio-dorsal (MD) and the anterior (A) group of nuclei as well as the cluster enclosing the centro-lateral and the lateral posterior nuclei along with the medial part of the Pulvinar (CL-LP-PuM). It can be seen that for all the subjects the Guiot points are always inside and/or on the border of the manual delineation, which furthermore is observed in the anterior-lateral part of the VLV cluster close to its lateral border.

**Figure 4:**
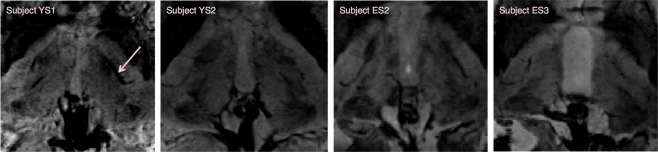
Illustration of the difficulties encountered for the manual delineation of the Vim regarding the image contrast on SWI acquired at 7T and the presence of blood vessels in the targeted area. We can observe that the contrast varies between subjects, but also between the two groups, young (here YS1 and YS2) versus the elderly (ES2 and ES3). The arrow for Subject YS1, illustrates the relatively big blood vessel passing through the left targeted thalamic region that prevented manual discrimination of the Vim. The presence of small vessels surrounding the Vim can be observed in each panel.

The Vims were identified and manually delineated in both hemispheres for eight out of nine subjects. In the ninth case, only the right Vim area was distinguishable, while on the left side, a blood vessel made it difficult to discriminate any contrast difference that could have corresponded to the Vim (Figs 3 and 4). Hence, in total, we had 17 manually delineated Vims.

The manual delineation was nonetheless challenging and time-consuming. In fact, the area of the Vim, appearing as a hyper-intensity structure, was directly detectable, but its peripheral borders were not always unquestionably discernible, due either to the lack of a consistent image intensity contrast or the presence of small blood vessels that sometimes could have been confounded with the surrounding nuclei. Moreover, the image intensity and its contrast variations differed between subjects and different age ranges (Fig. 4).

With the aim of providing reliable outlines, the rater chose not to include in the delineation the surrounding, more ambiguous darker regions. These outlines were then considered as a reference for the subsequently performed analyses.

The manually delineated volumes were in the range [35.5, 94.5] mm^3^ and occupied 0.4–1.5% of the total thalamic volume (Table 1).

**Table 1:** The calculated volume of the manually delineated Vim and the VLV cluster.

Volume	Vim								VLV			
	Manual delineation				Multi-atlas segmentation				Diffusion-based outline			
	Left		Right		Left		Right		Left		Right	
	mm^3^	normalized	mm^3^	normalized	mm^3^	normalized	mm^3^	normalized	cm^3^	normalized	cm^3^	normalized
Subject YS1	/^(*)^	/^(*)^	77.6	1%	/^(**)^	/^(**)^	/^(**)^	/^(**)^	1.1	15%	1	14%
Subject YS2	76.3	1%	82	1.1%	66.9	0.9%	71.6	1%	1.3	17%	1.3	18%
Subject YS3	83.3	1.5%	82.9	1.2%	28.5	0.5%	44.4	0.6%	1.1	18%	1.1	16%
Subject YS4	82.8	1.1%	73	1.1%	42.4	0.6%	42.4	0.6%	1	14%	0.9	15%
Subject YS5	67.2	1%	78.7	1%	43	0.6%	41.5	0.5%	1.2	19%	1	15%
Subject ES1	93	1.2%	94.5	1.3%	42.7	0.6%	23.7	0.3%	0.9	13%	0.8	13%
Subject ES2	58	0.5%	56.5	0.5%	19.4	0.2%	44.9	0.4%	1.4	14%	1.4	15%
Subject ES3	81.5	0.9%	65.5	0.8%	32.4	0.4%	9.7	0.1%	1.2	15%	1	14%
Subject ES4	71.4	1.1%	69.1	1%	20.2	0.3%	6.1	0.1%	0.7	13%	0.8	13%

The presence of a blood vessel made impossible the manual delineation of the Subject YS1’s right Vim (*) and therefore this subject was not considered in the analysis of multi-atlas segmentation (**).

### Comparison between the functional and the structural Vim definition

**Figure 5:**
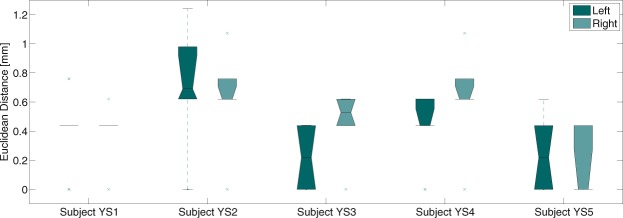
Boxplot showing the difference between the six targeting points obtained by the quadrilatere of Guiot for each young healthy subject respectively.

The obtained points from the individually repeated targeting with the quadrilatere of Guiot, a task performed only on the young cohort, were either overlapping or differing by one voxel for all subjects involved in this study (YS1-YS5). The maximum intra-subject difference was less than 1.3 mm, confirming the expected high reproducibility of this targeting (see the boxplots in Fig. 5).

The Guiot points were always inside or on the border of the manual delineation. Considering the geometrical subdivision (Fig. S1), for seven out of nine cases the Guiot points were in the ventral part of the manual delineation.

### Comparison with the automated methods

#### Automated 3T dMRI-based segmentation

For all nine subjects, the thalamic parcellation showed a similar segmentation pattern as previously reported for a larger cohort of 35 healthy subjects^31^ (Fig. 3). The segmented ventral latero-ventral (VLV) cluster, which is the outline of interest since it encloses the motor-related nuclei including the Vim^31^ (Fig. 1), represented the expected spatial extent and its volume was in the interval [0.74, 1.4] *cm*^3^ or 13–19% of the corresponding thalamic volume. Detailed values are reported in Table 1.

The VLV cluster consistently included the manual Vim outline. The size of the VLV was on average 15 times bigger than the manual outline (see Fig. 3). With respect to the spatial localization assessed via the geometrical subdivision (Fig. S1), for 16 out of 17 cases the manual Vim outline was found in the inferior VLV, in the last case being around the mid-plane. Furthermore, the manual outline was always next to the lateral VLV border and, in 15 cases, it was situated in the anterior/anterior-lateral part of this cluster. For the remaining two cases, the Vim outline was around the respective in-plane diagonal.

#### Multi-atlas segmentation

To assure an equal contribution from both hemispheres, the subject with no manual outline of the Vim (YS1) was not considered for building the multi-atlas framework, leading to eight subjects in total for this analysis. Hence, as we applied the leave-one-out (LOO) cross-validation, seven atlases were used per hemisphere.

**Figure 6:**
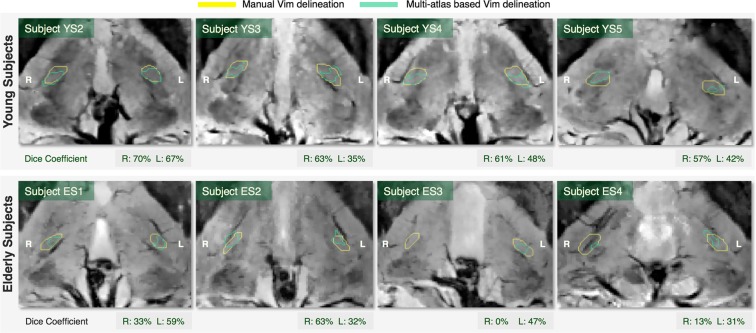
Visual comparison between the manual delineation and the multi-atlas outline of the Vim. The Dice coefficient estimates the overlap between the two outlines.

The multi-atlas segmentation outlines were fairly comparable to the manual delineation (Fig. 6), with the exception of one subject (ES3) right hemisphere where this methodology failed to provide an outline overlapping with the manual delineation. The calculated Dice overlap was 45.1+/−12.9% for the left and 45.0+/−26.4% for the right hemisphere. The individual Dice coefficients are given in Fig. 6 together with the visual representation of the outcome.

### Patients treated with Vim radiosurgery for essential tremor

We applied the same four approaches for defining the Vim, as performed in the healthy population, on two patients, with drug-resistant tremor, treated with left Vim RS for right-sided drug-resistant essential tremor. One was 86-years old and one 54-years old, referred to as P1 and P2, respectively. Indirect targeting was performed for both using the quadrilatere of Guiot. A dose of 130 Gy at the 100% isodose line was prescribed in each patient.

In the particular case of these patients, we observed that the Vim is also distinguishable on 7 Tesla imaging, fairly comparable as for the explored healthy population, previously described. The performed manual delineation presented volumes between 36.2 and 43.6 mm^3^. Furthermore, the thalamus parcellation provided again the expected segmentation pattern and the volumes of the VLV clusters were in the interval [0.77,1] cm^3^. Moreover, all four VLVs (left and right side of each patient) enclosed the manual delineation on the 7T SWI in their inferior anterio-lateral segment. Additionally, for three out of four Vims, the multi-atlas based segmentation gave outlines of volume between 19.5 and 40.8 mm^3^ and overlap with the corresponding manual delineation in the range between 31.4 and 51.6%. For the right side of P1, the outline enclosed only a few voxels and did not overlap the manual delineation.

**Figure 7:**
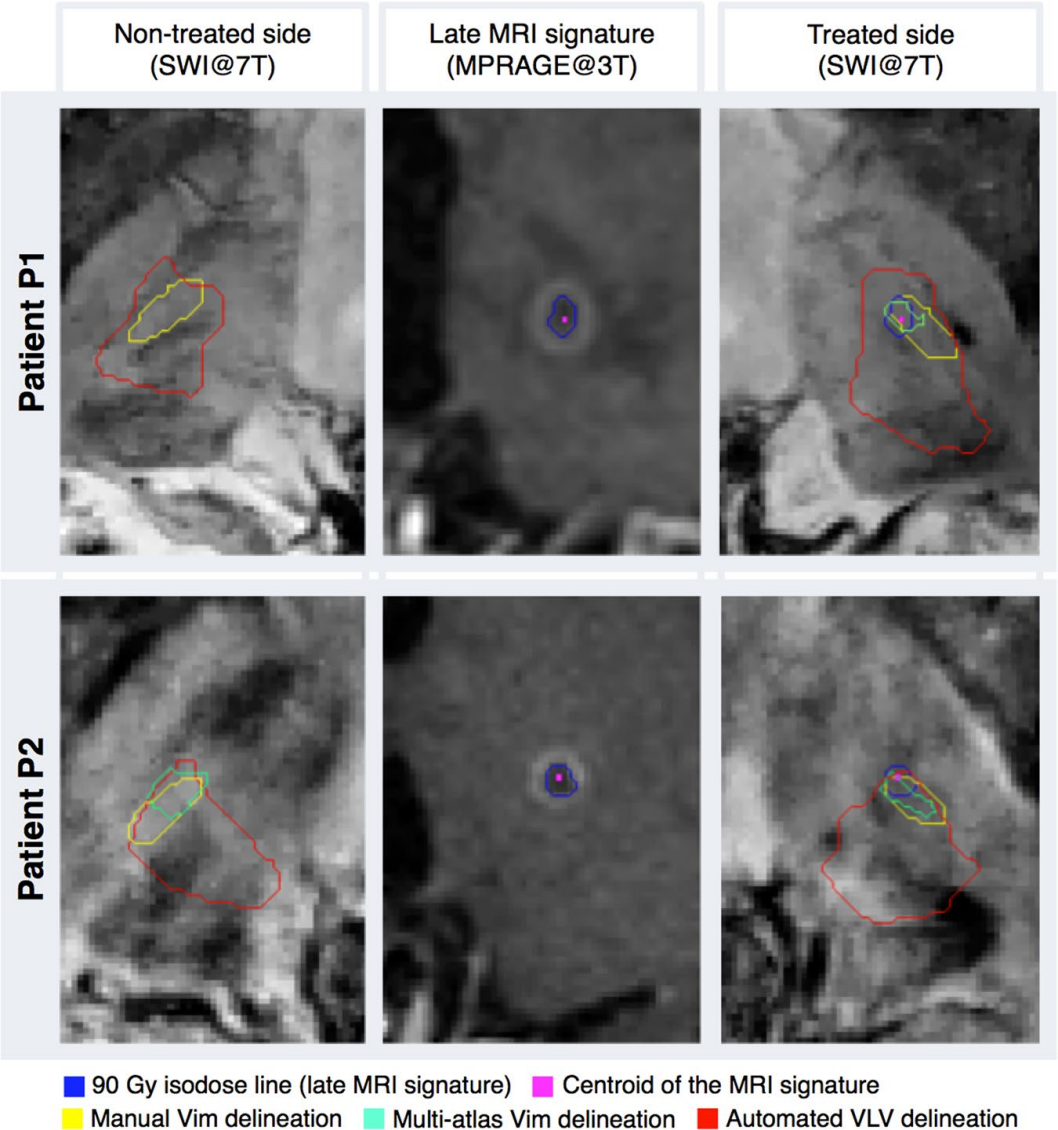
Illustration of the results from tremor patients. Both patients were treated on the left thalamus, with left Vim radiosurgery for right-sided drug-resistant essential tremor.

Moreover, the MR signature usually seen on the follow-up images after Vim RS (here at 3 and 9 months for P1 and P2 respectively), due to its delayed effect^32,33^, showed the classical “cockade aspect”, as in previously published reports^32,34^. The “cockade aspect”, as referred in the literature, contains both the peripheral ring of the contrast enhancement as well as the hypo-intensity, central area on T1 weighted images, all corresponding to the 90 Gy isodose line^34^ (refer to Fig. 7).

The MR signature overlapped with the anterior half of the manually delineated Vim. Furthermore, the centroid of this signature, which should represent the targeted point during the treatment, for P1, was in the ventral, while for P2 in the anterior Vim’s segment delineated geometrically (conforming to the Fig. S1 from the supplementary material). Additionally, these centroids were, in both cases, inside the multi-atlas segmentation outline. Overall results of the patients’ data are illustrated in Fig. 7.

## Discussion

This study presents extensive Vim-related analyses of 7T SWI whose enhanced image contrast in the central brain allows a direct visualization of the thalamic subparts. This advantage of directly distinguishing the Vim was demonstrated here for data coming from both healthy volunteers representing broad age intervals and drug-resistant essential tremor patients. Additionally, having the manual delineation of the Vim done on 7T SWI, the study also compares the current indirect clinical targeting and the existing automated image-segmentation techniques against this outline. Hence, the presented work provides a reinforced knowledge characterizing the Vim.

In the young healthy-population data, we observed that the Guiot’s targeting points, are for 77.8% of the cases in the geometrically delineated ventral part of the manual Vim delineation based on 7T SWI. These observations were in line with the findings from the patients’ data, where the MRI signature appearing on the follow-up images was enclosed in the anterior half of the manual outline. Although the Guiot targeting was not performed on the healthy elderly population, observing the high robustness of the outcome in young subjects and patients, we assume that the same findings would be found in any additional data.

The Guiot targeting allocates only a single point and, as a consequence, is limited in providing a full structural discrimination of the Vim. In this context, our findings, obtained from 11 Vims in total, indicate a confined localization of the clinically aimed points inside a structural outline. Hence, the ventral part of the Vim should be further investigated in terms of connectivity and function for potentially obtaining advanced target-related insights. Moreover, an extension of this study on a larger dataset could possibly lead to a robust prediction of the neurosurgical target emplacement inside the Vim’s structural borders.

Our results also suggested that the manual delineation, 15 times smaller than the size of the VLV, is mainly enclosed (95.2% of the studied cases) in the inferior anterior-lateral division of the VLV cluster, which is anatomically relevant. Moreover, such findings reveal a constrained area that most likely corresponds to the Vim within a region automatically delineated by computer-assisted image-analysis techniques, which has not been demonstrated previously. Future developments could exploit this new spatial prior knowledge as a constraint for further subdivision of the VLV cluster, for instance based again on dMRI or in combination, if available, with 7T SWI features.

The proposed multi-atlas segmentation framework shows promising potential of delineating the Vim area in automated manner. Although the outcome tends to slightly underestimate the volume of the Vim, in both patients’ data it overlapped with the late MRI signature enclosing the centroid of this region. In the healthy population data, as well, the multi-atlas outline is relatively small but, however, fairly acceptable, mainly in terms of identifying the Vim area. With regard to the quantitative estimation of the overlap, a Dice Coefficient greater than 70%, is commonly considered as a good match. Nevertheless, this value has been established for evaluating the segmentation accuracy for relatively big structures. In fact, Dice is more sensitive to the variation of edge voxels than the inner ones and therefore, for small and elongated structures as the Vim, one could argue that such measure could bias the estimation of the overlap^35^.

We assume that the presented promising potential of the proposed multi-atlas segmentation is closely related to the use of SWI-intensity instead of T1w or T2w, lacking in intrathalamic contrast variation. Consequently, when 7T SWI is available, this approach could be used as an alternative or as an initialization of a subject-related segmentation of the Vim, either manual or automated one. Nevertheless, we expect improvement of the presented findings with an extension/adaptation of the number of employed atlases to its optimum^36^.

Although our main aim focuses on the Vim, this study also provides validation of the automated dMRI-based clustering against the directly visible SWI regions inside the thalamus. For example, in SWI the Pulvinar appears as the most-posterior distinguishable thalamic feature very dark laterally and brighter next to the ventricles (Fig. 3). Accordingly to previously reported findings^31^, in Fig. 3 we see that the green Pu-cluster contour delineates mainly the darker nuclei part and a portion of medial Pulvinar (the brighter part) is enclosed in the cluster representing the centro-lateral and the lateral posterior nuclei along with the medial part of the Pulvinar (CL-LP-PuM). Furthermore, the spatial extent of the medio-dorsal (MD) cluster coincides in general to the equivalent group of nuclei. All these findings support, above all, the spatial distribution of the VLV and thereupon, our findings related to this cluster.

The manual delineation on the 7T SWI data is however a time-consuming task. In fact, the region corresponding to the Vim is, as shown by our data, visually directly distinguishable both in healthy controls and in patients. However, the difficulties arise in the presence of blood vessels or a low local image contrast, both of them making more challenging the precise discrimination of the peripheral borders of this nucleus. Hence, the provided outline encloses only the region uncontestably corresponding to the Vim. Or, in other words, it represents well the Vim, but tends towards a slight underestimation of its extent. This could also be deduced from the calculated volumes (Table 1), which are near the lower limits of the known range^3^.

Moreover, despite the fact that no differences were noticed between the SWI acquired from the patients and the elderly healthy volunteers, we however observed inconsistency in terms of the contrast variation between the cohorts representing different age ranges. Namely, the elderly subjects presented poorer contrast, which increased the difficulty in immediate identification of the Vim in those cases (Fig. 4). Nevertheless, the intrathalamic contrast remains globally similar and adequate for outlining the supposed Vim area.

Supplementary SWI analysis indicated that the image intensity contrast could also depend on the head orientation inside the scanner. This could be caused by anisotropic contributions to the local tissue susceptibility. For instance, white matter fibres have significantly anisotropic susceptibility^37^. Depending on the head orientation relative to the static MR field, the local susceptibility-based perturbations of the field may differ, and the resulting effects may thereby show significant changes in contrast. Anisotropic voxels may also add different partial volume effects depending on the head orientation.

The multi-atlas segmentation could also be troubled by the mentioned contrast-related drawbacks. We think that the relatively small volumes obtained from the multi-atlas segmentation are related on one side to eventual minor registration errors and on the other side to the Joint Label Fusion method we apply a posteriori, as both registration and fusion can be disturbed by the random presence of vessels. Moreover, the stated drawbacks could also be associated with the non-symmetrical tendency of the multi-atlas outcome. We assume as well that using a bigger cohort will not only allow finding the optimum number of atlases, but it could also decrease the left-right asymmetry between the multi-atlas outlines.

Regarding the stated limitations, further improvements including contrast standardization and vessel removal^38^ should be addressed in order to make 7T SWI an even more powerful and robust tool for Vim discrimination. Additionally, such standardization could be beneficial for further investigating if the 7T SWI is capable to capture eventual anatomical differences between the controls and the patients’ data.

The SWI sequences are also prone to spatial distortions, but as Duchin *et al*. have already demonstrated^39^, the central brain area and therefore the thalamus as well, are negligibly affected by those distortions. However, to compensate for the peripheral spatial distortions observed on the 7T data, an affine transform was employed to match them to the corresponding 3T images. Additionally, considering the motion, even though the tremor patients are more prone to such artefacts, there were no particular differences between the healthy subjects and the patients in terms of the acquired data and the obtained findings.

Although the number of included clinical cases is low, the findings observed in the patients are in line with those from the explored healthy population. Moreover, as both of the patients are presenting a good clinical outcome, their contribution to this work becomes even more valuable.

Nevertheless, in line with the pioneer work of Abosch *et al*.^21^, which is mainly in the scope of defining the subthalamic nucleus and other basal ganglia structures on 7T SWI, by exploring a larger dataset from healthy subjects and, moreover, extending it to clinical cases, the present study demonstrates even further the high potential of SWI acquired at 7T to provide more precise characterization of the Vim as a structure than the other existing methods currently used to define this nucleus. Furthermore, our comparison analyses, where the manual Vim delineation done on 7T SWI was taken as a reference, indicate two new promising approaches for an automated localization and delineation of the Vim: 1) the localization likelihood inside the cluster obtained from the robust and reproducible 3T dMRI-based thalamic parcellation and 2) the multi-atlas segmentation exploring the 7T SWI information.

Further studies on a larger dataset and including more raters for the manual delineation should confirm the reported findings. Improvements could also include multi-atlas segmentation based on 7T SWI only. Moreover, given the potential of the automated dMRI-based segmentation, we assume that combining it with the complementary 7T SWI information would further lead to a more accurate Vim outline based only on the individual anatomy.

## Methods

Local institutional review board named *Commission cantonale d'éthique de la recherche sur l'être humain (CER-VD)* approved the study and all participants gave written informed consent. All the analyses performed in the presented work were done in accordance with the relevant guidelines and regulation stated in the obtained approval from CER-VD with protocol number 165/15.

### Image acquisitions

The acquisitions at 3T (Prisma and 3T TIM-Trio SIEMENS scanner, 32-channel head coil) included the standard clinical Vim RS protocol: coronal T2-weighted (T2w), T2-weighted Constructive Interference Steady State (CISS)/Fiesta, T1-weighted (T1w, MPRAGE) and diffusion weighted imaging (DWI) acquired with 72 gradient directions and b = 1000 *s/mm*^2^. At 7T (68 *cm-wide bore* MRI system (SIEMENS Medical Solutions), 32-channel head coil (Nova Medical)) we acquired T1w MP2RAGE^40^ and axial SWI. The acquisition parameters are given in Table 2.

**Table 2:** MRI protocol. The abbreviation YS denotes the parameters used for the acquisitions from the young cohort, ES from the elderly one, and P for the patients, where they differed between them.

Sequence	3T T2-w	3T T2-w CISS	3T MPRAGE	3T DWI	7T MP2RAGE	7T SWI
Scanning Machine (Siemens)	YS: Tim Trio	YS: Tim Trio	YS: Tim Trio;ES,P: Prisma	YS: Tim Trio; ES,P: Prisma	Magnetom (head only)	Magnetom (head only)
Resolution, mm^3^	YS: 0.5 × 0.5 × 1.0	YS: 0.4 × 0.4 × 0.4	1.0 × 1.0 × 1.0	2.2 × 2.2 × 2.2	YS1, YS2, ES2, P1, P2: 0.8 × 0.8 × 0.8; Others: 0.6 × 0.6 × 0.6	0.375 × 0.375 × 1.0
Axial matrix size	YS: 512 × 512	YS: 320 × 320	256 × 256	98 × 98	YS1, YS2, ES3, P1, P2: 240 × 256; Others: 256 × 320	512 × 512
Slice/partitions	YS: 160	YS: 80	YS: 160; ES, P: 192	YS: 52; ES, P: 62	YS1, YS2, ES3, P1, P2: 176; Others: 320	72
Repetition time, ms	YS: 3200	YS: 6.18	2300	YS: 6300; ES, P: 7100	6000	28
Echo time, ms	YS: 402	YS: 2.75	YS: 2.98; ES, P: 2.03	84	YS: 2.64; ES: 2.05	20
Inversion time, ms	/	/	900	/	800/2700	/
Flip angles, degrees	YS: 120	YS: 49	9	90	7/5	10
Target	Whole Brain	Thalamus	Whole Brain	Whole Brain	Whole Brain	Thalamus

As an added value, to evaluate the appearance of the MR signature after Vim RS, Gadolinium-enhanced MPRAGE was acquired every three months for the two patients, with drug-resistant essential tremor. The acquisition was done at 3T with similar parameters, as for the preoperational 3T MPRAGE. The first patient (P1) showed decreased tremor amplitude on the right, contralateral hand at four months, with further complete tremor arrest at six months, while the second patient (P2), had major tremor decrease at 3 months after the procedure. This type of clinical answer is common after Vim RS for tremor^18^.

The subjects were carefully immobilized with head positioning pads to minimize motion and were instructed to avoid moving during the individual image acquisitions.

### Common image space

The analyses, for each subject respectively, were performed in the individual anterior commissure - posterior commissure (AC-PC) image space.

For the young population, we first transformed the T2w into the AC-PC space by employing 3D Slicer^41^ and choosing manually 30 brain mid-plane points. The resulting image was then used as a reference for the AC-PC alignment of the remaining sequences. More precisely, for each 3T contrast, with the exception of the DWI, we performed a rigid-body transformation (six degrees of freedom, DOF).

For the dMRI data, we registered the fractional anisotropy (FA) map to the MPRAGE with a non-linear transform using FSL FNIRT^42,43^, to simultaneously account for the echo planar imaging (EPI) distortions^44^.

Moreover, the observed distortions in the frontal and the parietal cortex of the 7T data were compensated with a 12-DOF linear transform between both skull-stripped 7T MP2RAGE and 3T MPRAGE. This transform was applied to the SWI rigidly (6-DOF) aligned beforehand to the corresponding MP2RAGE.

All registrations^45^ were performed with 100,000 voxel samples and Mattes Mutual Information as the cost function. The outcome quality was assessed by visual inspection of the matching between the ventricles.

Figure S2 in the supplementary material summarizes the registration process. After being transformed to the AC-PC space, all images were resampled to the T2 CISS spatial resolution (0.4 × 0.4 × 0.4 mm^3^) using linear interpolation.

Since the 3T images of the elderly cohort were acquired directly in AC-PC space, the AC-PC transformation was not required in these cases, but correspondence between the respective images was however performed according to the previously described flow for the young cohort. After being transformed, all the images were interpolated to the voxel size of 0.5 × 0.5 × 0.5 mm^3^ defined by the T1 template in MNI space^46^.

### Manual Vim delineation on 7T SWI

SWI combines contrast from differences in effective transverse signal relaxation (T2*) and in MR signal phase, both strongly affected by the magnetic susceptibility and the underlying tissue geometry^23,24^. Accordingly, SWI has shown increased sensitivity with respect to standard T1- or T2*-weighted imaging for visualizing deoxygenated blood vessels, vascular and axonal lesions, as well as calcium and iron depositions^47^ and it allows better visualization of certain deep brain structures, including the thalamic subparts. Particularly, the Vim appears as a well-distinguishable hyper-intensity structure surrounded by darker regions^21^ (Fig. 1a). This is especially evident at higher magnetic field strengths, such as 7T, where the susceptibility-dependent contrast is more accentuated, and moreover, sub-millimeter spatial resolution can be easily achieved.

The manual delineation of the Vim on 7T SWI was done by an experienced neurosurgeon (CT) and was primarily based upon the previous observations of Abosch *et al*.^21^ and the Schaltenbrand and Wahren atlas^48^ also used as reference in the mentioned study.

The thalamic lateral border and the internal capsule are well distinguishable on the 7T SWI. Referring to the Shaltenbrand’s atlas, the Pulvinar as well is directly recognizable in the axial SWI plane (in green, Fig. 1a). Superior to it, again in axial plane, is the ventro-caudalus nucleus (Vc) appearing as a dark region (Fig. 1a, bright-violet) immediately posterior to a narrower zone with brighter image intensity that is considered as Vim (Fig. 1a, yellow). Above it, ventro-odalis (Vo) appears (Fig. 1a, bright-blue) as another dark area defining the superior border of the Vim. Additionally, the visualization of the medio-dorsal group allows the identification of the medial Vim border.

### Clinical and research methods

#### Quadrilatere of Guiot

The quadrilatere of Guiot^2,15^ is defined upon anatomical landmarks, including AC, PC, thalamus height, and third-ventricle lateral wall, all easily recognizable on T2 CISS. The final position is the Vim’s anterio-inferior part, at 11 mm from the third-ventricle lateral border. An experienced neurosurgeon (CT) performed this targeting in MITK 3M3 (German Cancer Research Center, Heilderberg, Germany) on the five young subjects (YS1-YS5).

As the targeting procedure was done outside of regular treatment-planning software, the quadrilatere of Guiot was built independently six times for each subject, bilaterally and blindly with the aim of assessing its reproducibility. A sphere with 2 mm radius was drawn around each Guiot point to simulate both the 90 Gy isodose line and the *contrast-enhanced* area^49^ visualized on follow-up MRI if a radiosurgical thalamotomy^50^ would have been applied in the presented cases.

#### Automated 3T dMRI-based segmentation

Several research groups explored the dMRI advantages for automated thalamic subdivision^31,51–58^. The initial approaches are mainly based on diffusion tensor images i.e. a coarse diffusion representation. Recently, our group developed an approach^31^ that relies on a more detailed diffusion feature - the Spherical Harmonic (SH) representation of the Orientation Distribution Functions (ODFs) and outperforms the state-of-the-art methods by providing robust and reproducible segmentation pattern for a large dataset and different diffusion sequences. Our approach subdivides the thalamus into seven groups of nuclei corresponding to the anatomy in Morel’s atlas^59^. One of the segmented groups of nuclei is the ventral latero-ventral cluster (VLV) enclosing the motor-related nuclei including the Vim (Fig. 1).

The pre-processing of the dMRI data included data denoising^60–62^, bias field^43,63^, motion^64^ and eddy current correction^65^. The SH coefficients of the constant solid angle ODFs of maximum order 6 were calculated using the FSL’s qboot function^31^.

The thalamic masks were initially obtained from the Freesurfer parcellation^66,67^ on each MPRAGE and redefined, as described by Battistella *et al*.^31^, by eliminating the voxels having FA value greater than 0.55 or exceeding 5% probability to belong to the cerebrospinal fluid. The refined masks were further compared to the thalamic borders appearing more evident in SWI and consequently, for providing even more precise thalamic outline, several voxels were manually refined.

The thalamic subdivision was completed in the diffusion space of each subject and the resulting clusters were brought in the AC-PC space.

#### Multi-atlas segmentation

One widely used state-of-the-art segmentation technique is the atlas-based registration, if an atlas is available. Having achieved the manual Vim outline, we considered each one of them as an atlas within a multi-atlas segmentation framework.

The framework was built in leave-one-out scenario, meaning that for each subject-*target* its own Vim outlines were not considered as atlases, but only those from the remaining subjects. The atlas registration was done in 2 steps.

First, with an affine transform^45^ we matched the respective MPRAGEs and we applied it to the corresponding SWI. Second, we performed a non-linear registration for a local correspondence using only the SWI as it gives more distinguishable thalamic features than the T1w, thus potentially allowing more accurate matching between the thalamic nuclei.

To this end, we defined a volume of interest (VOI) surrounding the thalamus in which, in order to standardize the SWI intensities among all the subjects, we performed a histogram matching proposed by Nyiul *et al*.^68^. The standard scale of this intensity matching method was built upon the histogram deciles from the SWI-VOI of the elderly population and was then used as the reference scale for the one-to-one intensity mapping of all the SWI-VOIs.

Non-rigid BSpline transform using maximum displacement of 1 mm^45^ was computed in between the respective SWI-VOIs. The combination of both linear and non-linear transform was applied to the Vim outlines respectively and the final multi-atlas segmentation outcome was obtained by employing the Joint Label Fusion method with corrective learning^69^ with the default parameters.

## Supplementary information

Supplementary material: Fig. S1 is givning a schematic representation of the geometrical ROIs separation, while Fig. S2 gives an overview of the registrations applied for transforming the individual data into common image space

## Data Availability

All the original images used in the presented study and the corresponding manual delineation of both left and right Vim are uploaded in Zenodo (10.5281/zenodo.1438358).

## References

[1] Massion J (1976). The thalamus in the motor system. Appl Neurophysiol.

[2] Guiot G, Hardy J, Albe-Fessard D (1962). Precise delimitation of the subcortical structures and identification of thalamic nuclei in man by stereotactic electrophysiology. Neurochirurgia.

[3] Tuite, P. J. & Dagher, A. *Magnetic resonance imaging in movement disorders: a guide for clinicians and scientists*. (Cambridge University Press, 2013).

[4] Benabid AL (1991). Long-term suppression of tremor by chronic stimulation of the ventral intermediate thalamic nucleus. Lancet.

[5] Blond S (1992). Control of tremor and involuntary movement disorders by chronic stereotactic stimulation of the ventral intermediate thalamic nucleus. Journal of neurosurgery.

[6] Goldman MS, Ahlskog JE, Kelly PJ (1992). The symptomatic and functional outcome of stereotactic thalamotomy for medically intractable essential tremor. Journal of neurosurgery.

[7] Schuurman PR (2000). A comparison of continuous thalamic stimulation and thalamotomy for suppression of severe tremor. The New England journal of medicine.

[8] Kondziolka D (2008). Gamma Knife thalamotomy for essential tremor. Journal of neurosurgery.

[9] Ohye, C. *et al*. Gammaknife thalamotomy for Parkinson’s disease and essential tremor: A prospective multicenter study. *Neurosurgery*, 10.1227/NEU.0b013e3182350893 (2011). 10.1227/NEU.0b013e3182350893 21904267

[10] Young RF, Li F, Vermeulen S, Meier R (2010). Gamma Knife thalamotomy for treatment of essential tremor: long-term results. J Neurosurg.

[11] Elias WJ (2013). A pilot study of focused ultrasound thalamotomy for essential tremor. N Engl J Med.

[12] Lipsman N (2013). MR-guided focused ultrasound thalamotomy for essential tremor: a proof-of-concept study. The Lancet. Neurology.

[13] Guiot G (1962). Interpretation of the effects of thalamus stimulation in man by isolated shocks. C R Hebd Seances Acad Sci.

[14] Witjas T (2015). A prospective single-blind study of Gamma Knife thalamotomy for tremor. Neurology.

[15] Guiot G, Brion S, Akerman M (1961). Stereotaxic anatomy of the internal pallidum, of the thalamus and of the internal capsule. Studies of the individual variations. II. Ann Chir.

[16] Albe-Fessard D (1966). Electrophysiological studies of some deep cerebral structures in man. Journal of the neurological sciences.

[17] Ohye, C. *et al*. Gamma knife thalamotomy for Parkinson disease and essential tremor: a prospective multicenter study. *Neurosurgery* **70**, 526–535, discussion 535–526, 10.1227/NEU.0b013e3182350893 (2012). 10.1227/NEU.0b013e3182350893 21904267

[18] Tuleasca C (2018). Clinical response to Vim’s thalamic stereotactic radiosurgery for essential tremor is associated with distinctive functional connectivity patterns. Acta neurochirurgica.

[19] Witjas T, Carron R, Eusebio A, Azulay JP, Regis J (2013). Gammaknife Thamamotomy for Intractable Tremors: Clinical Outcome and Correlations with Neuroimaging Features (P05.032). Neurology.

[20] King NKK (2017). Anatomic Targeting of the Optimal Location for Thalamic Deep Brain Stimulation in Patients with Essential Tremor. World neurosurgery.

[21] Abosch, A., Yacoub, E., Ugurbil, K. & Harel, N. An assessment of current brain targets for deep brain stimulation surgery with susceptibility-weighted imaging at 7 tesla. *Neurosurger*y **6**7, 1745–1756; discussion 1756, 10.1227/NEU.0b013e3181f74105 (2010). 10.1227/NEU.0b013e3181f74105 PMC3124849 21107206

[22] Lenglet C (2012). Comprehensive *in vivo* mapping of the human basal ganglia and thalamic connectome in individuals using 7T MRI. PloS one.

[23] Haacke EM, Mittal S, Wu Z, Neelavalli J, Cheng YC (2009). Susceptibility-weighted imaging: technical aspects and clinical applications. AJNR. American journal of neuroradiology.

[24] Haacke EM, Xu Y, Cheng YC, Reichenbach JR (2004). Susceptibility weighted imaging (SWI). Magnetic resonance in medicine: official journal of the Society of Magnetic Resonance in Medicine/Society of Magnetic Resonance in Medicine.

[25] Schmidt MA (2017). Ultra high-field SWI of the substantia nigra at 7T: reliability and consistency of the swallow-tail sign. BMC neurology.

[26] Alkemade A (2017). Comparison of T2*-weighted and QSM contrasts in Parkinson’s disease to visualize the STN with MRI. PloS one.

[27] Liu T (2013). Improved subthalamic nucleus depiction with quantitative susceptibility mapping. Radiology.

[28] Rasouli J (2018). Utilization of Quantitative Susceptibility Mapping for Direct Targeting of the Subthalamic Nucleus During Deep Brain Stimulation Surgery. Operative Neurosurgery.

[29] Deistung A (2013). Toward *in vivo* histology: a comparison of quantitative susceptibility mapping (QSM) with magnitude-, phase-, and R2*-imaging at ultra-high magnetic field strength. NeuroImage.

[30] Dice LR (1945). Measures of the Amount of Ecologic Association Between Species. Ecology.

[31] Battistella G (2017). Robust thalamic nuclei segmentation method based on local diffusion magnetic resonance properties. Brain structure & function.

[32] Tuleasca C, Regis J, Levivier M (2018). Essential Tremor. The New England journal of medicine.

[33] Tuleasca C (2017). Assessing the clinical outcome of Vim radiosurgery with voxel-based morphometry: visual areas are linked with tremor arrest!. Acta neurochirurgica.

[34] Regis J, Carron R, Park M (2010). Is radiosurgery a neuromodulation therapy?: A 2009 Fabrikant award lecture. Journal of neuro-oncology.

[35] Deeley MA (2011). Comparison of manual and automatic segmentation methods for brain structures in the presence of space-occupying lesions: a multi-expert study. Physics in medicine and biology.

[36] Van de Velde J (2016). Optimal number of atlases and label fusion for automatic multi-atlas-based brachial plexus contouring in radiotherapy treatment planning. Radiation oncology.

[37] Wharton S, Bowtell R (2015). Effects of white matter microstructure on phase and susceptibility maps. Magnetic resonance in medicine: official journal of the Society of Magnetic Resonance in Medicine/Society of Magnetic Resonance in Medicine.

[38] Jorge, J. *et al*. In *Annual Meeting and Exhibition of the International Society for Magnetic Resonance in Medecine (ISMRM)* (Paris, France, 2018).

[39] Duchin Y, Abosch A, Yacoub E, Sapiro G, Harel N (2012). Feasibility of using ultra-high field (7 T) MRI for clinical surgical targeting. PloS one.

[40] Marques JP (2010). MP2RAGE, a self bias-field corrected sequence for improved segmentation and T1-mapping at high field. NeuroImage.

[41] Fedorov A (2012). 3D Slicer as an image computing platform for the Quantitative Imaging Network. Magnetic Resonance Imaging.

[42] Andersson, J. L. R., Jenkinson, M. & Smith, S. Non-linear registration aka Spatial normalization (2007).

[43] Smith SM (2004). Advances in functional and structural MR image analysis and implementation as FSL. NeuroImage.

[44] Irfanoglu MO, Walker L, Sarlls J, Marenco S, Pierpaoli C (2012). Effects of image distortions originating from susceptibility variations and concomitant fields on diffusion MRI tractography results. NeuroImage.

[45] Johnson H.J., Harris G. & Williams, K. BRAINSFit: Mutual Information Registrations of Whole-Brain 3D Images, Using the Insight Toolkit. *The Insight Journal* (2007).

[46] Fonov VS, Evans AC, McKinstry RC, Almli CR, Collins DL (2009). Unbiased nonlinear average age-appropriate brain templates from birth to adulthood. NeuroImage.

[47] Mittal S, Wu Z, Neelavalli J, Haacke EM (2009). Susceptibility-weighted imaging: technical aspects and clinical applications. AJNR. American journal of neuroradiology.

[48] Schaltenbrand, G. & Wahren, W. *Atlas For Stereotaxy of The Human Brain With Guide to The Atlas For Stereotaxy of The Human Brain*. (Thieme, 1977).

[49] Tuleasca C (2017). Deep brain stimulation after previous gamma knife thalamotomy of the Vim for essential tremor is feasible! Clinical, electrophysiological and radiological findings. Acta neurochirurgica.

[50] Campbell AM, Glover J, Chiang VL, Gerrard J, Yu JB (2015). Gamma knife stereotactic radiosurgical thalamotomy for intractable tremor: a systematic review of the literature. Radiotherapy and oncology: journal of the European Society for Therapeutic Radiology and Oncology.

[51] Behrens TE (2003). Non-invasive mapping of connections between human thalamus and cortex using diffusion imaging. Nature neuroscience.

[52] Jonasson L (2007). A level set method for segmentation of the thalamus and its nuclei in DT-MRI. Signal Process.

[53] Mang SC, Busza A, Reiterer S, Grodd W, Klose AU (2012). Thalamus segmentation based on the local diffusion direction: a group study. Magnetic resonance in medicine: official journal of the Society of Magnetic Resonance in Medicine/Society of Magnetic Resonance in Medicine.

[54] O’Muircheartaigh J (2011). Clustering probabilistic tractograms using independent component analysis applied to the thalamus. NeuroImage.

[55] Rittner, L., Lotufo, R. A., Campbell, J. & Pike, G. B. Segmentation of Thalamic Nuclei Based on Tensorial Morphological Gradient of Diffusion Tensor Fields. *I S Biomed Imaging*, 1173–1176, 10.1109/Isbi.2010.5490203 (2010).

[56] Stough, J. *et al*. In *Medical Image Computing and Computer-Assisted Intervention – MICCAI2014* Vol. 8675 *Lecture Notes in Computer Science* (eds Polina Golland *et al*.) Ch. 22, 169–176 (Springer International Publishing, 2014).

[57] Wiegell MR, Tuch DS, Larsson HB, Wedeen VJ (2003). Automatic segmentation of thalamic nuclei from diffusion tensor magnetic resonance imaging. NeuroImage.

[58] Ziyan U, Tuch D, Westin CR (2006). Segmentation of thalamic nuclei from DTI using spectral clustering. Lect Notes Comput Sc.

[59] Morel A, Magnin M, Jeanmonod D (1997). Multiarchitectonic and stereotactic atlas of the human thalamus. The Journal of comparative neurology.

[60] Tournier JD, Calamante F, Connelly A (2012). MRtrix: Diffusion tractography in crossing fiber regions. Int J Imag Syst Tech.

[61] Veraart J, Fieremans E, Novikov DS (2016). Diffusion MRI noise mapping using random matrix theory. Magnetic resonance in medicine: official journal of the Society of Magnetic Resonance in Medicine/Society of Magnetic Resonance in Medicine.

[62] Veraart J (2016). Denoising of diffusion MRI using random matrix theory. NeuroImage.

[63] Zhang Y, Brady M, Smith S (2001). Segmentation of brain MR images through a hidden Markov random field model and the expectation-maximization algorithm. IEEE transactions on medical imaging.

[64] Leemans A, Jones DK (2009). The B-Matrix Must Be Rotated When Correcting for Subject Motion in DTIData. Magnet Reson Med.

[65] Andersson JL, Sotiropoulos SN (2016). An integrated approach to correction for off-resonance effects and subject movement in diffusion MR imaging. NeuroImage.

[66] Fischl B (2002). Whole brain segmentation: automated labeling of neuroanatomical structures in the human brain. Neuron.

[67] Fischl B (2004). Automatically parcellating the human cerebral cortex. Cerebral cortex.

[68] Nyul LG, Udupa JK, Zhang X (2000). New variants of a method of MRI scale standardization. IEEE transactions on medical imaging.

[69] Wang H, Yushkevich PA (2013). Multi-atlas segmentation with joint label fusion and corrective learning-an open source implementation. Frontiers in neuroinformatics.

