# Supplementary material for: Comparison of MRI-based automated segmentation methods and functional neurosurgery targeting with direct visualization of the Ventro-intermediate thalamic nucleus at 7T
Source: Sci Rep. 2019 Feb 4;9:1119. doi: 10.1038/s41598-018-37825-8 (PMC6361927; doi:10.1038/s41598-018-37825-8)
Supplement: Supplementary file 1 — Supplementary material: Fig. S1 is givning a schematic representation of the geometrical ROIs separation, while Fig. S2 gives an overview of the registrations applied for transforming the individual data into common image space [file 41598_2018_37825_MOESM1_ESM.pdf]

# Comparison of MRI-based automated segmentation methods and functional neurosurgery targeting with direct visualization of the Ventro-intermediate thalamic nucleus at 7T

Elena Najdenovska, PhD, Constantin Tuleasca, MD, João Jorge, PhD, Philippe Maeder, MD, José P. Marques, PhD, Timo Roine PhD, Daniel Gallichan, PhD, Jean-Philippe Thiran PhD, Marc Levivier MD, PhD and Meritxell Bach Cuadra PhD

## SUPLEMENTARY MATERIAL

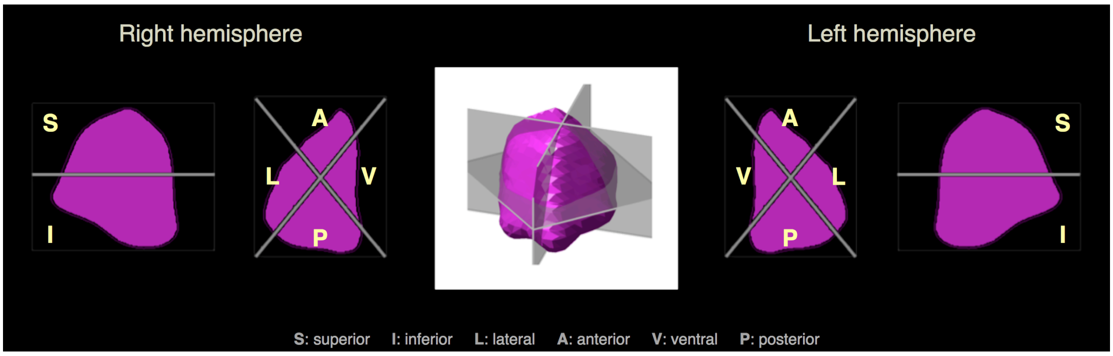


**Figure S1**. Schematic representation of the ROIs separation in 8 regions in 2D and 3D view (middle). The labelling of the subparts is done accordingly to the hemispheres to which the ROI belongs.

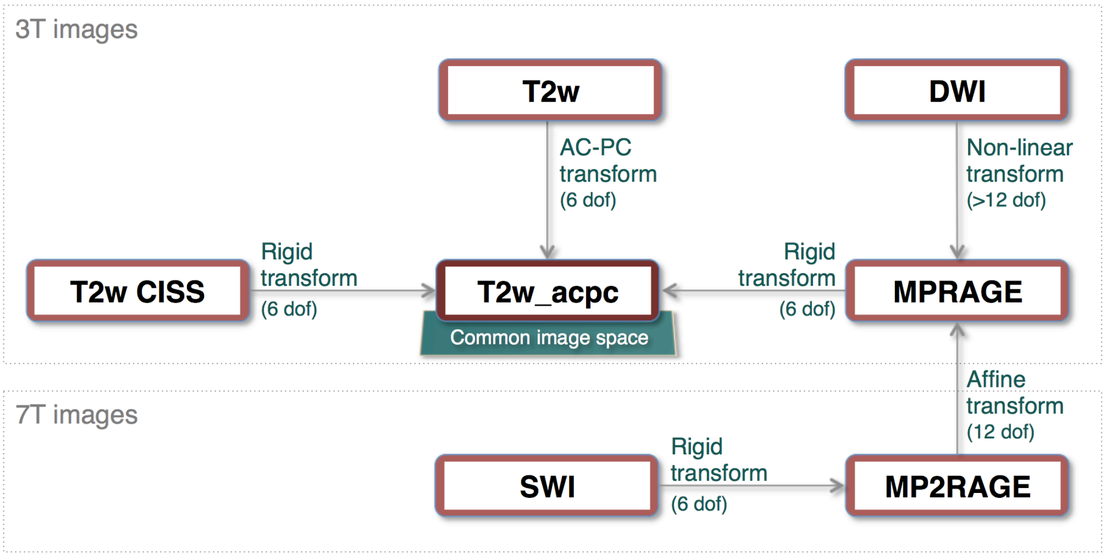


**Figure S2**. Schematic overview of the registrations applied for transforming the individual data into common image space.
